# Reducing Aflatoxin Accumulation in Maize: Development and Performance of a Novel Biological Input

**DOI:** 10.3390/toxins18010049

**Published:** 2026-01-17

**Authors:** Paloma Rhein, Marianela Bossa, María del Pilar Monge, Diego Giovanini, César Alfredo Barbero, Sofía Noemí Chulze, María Laura Chiotta, María Silvina Alaniz-Zanon

**Affiliations:** 1 Instituto de Investigación en Micología y Micotoxicología (IMICO), Consejo Nacional de Investigaciones Científicas y Técnicas (CONICET)-Universidad Nacional de Río Cuarto (UNRC), Ruta Nac. 36, Km 601. Río Cuarto, Córdoba 5800, Argentina; prhein@exa.unrc.edu.ar (P.R.); mbossa@exa.unrc.edu.ar (M.B.); mmonge@exa.unrc.edu.ar (M.d.P.M.); dgiovanini@ayv.unrc.edu.ar (D.G.); schulze@exa.unrc.edu.ar (S.N.C.); mchiotta@exa.unrc.edu.ar (M.L.C.); 2 Instituto de Investigaciones en Tecnologías Energéticas y Materiales Avanzados (IITEMA), CONICET-UNRC, Ruta Nac. 36, Km 601. Río Cuarto, Córdoba 5800, Argentina; cbarbero@exa.unrc.edu.ar

**Keywords:** aflatoxins, *Aspergillus flavus*, biocontrol, bioformulates, starch-based polymer, maize

## Abstract

Aflatoxin contamination of maize by Aspergillus section Flavi constitutes a major health and economic concern. While biological control using non-toxigenic strains has proven effective, the increasing global food demand underscores the need for alternative carrier materials to replace seeds and grains. The aims of the present study were (1) to develop an innovative macroporous starch polymer in which the biocontrol agent can grow and be transported to fields where the bioformulate is applied, and (2) to evaluate the effectiveness of this new formulate in reducing AF contamination in maize kernels in field trials, in comparison with the traditional formulate based on long-grain rice as a substrate. Several methods and different starch sources were tested, and the formulation consisting of 10% maize starch, 0.5% citric acid, 3% sucrose, 0.3% urea, and distilled water was the most effective. Furthermore, this bioformulate demonstrated a performance comparable to that of the traditional long-grain rice-based formulation, reducing AF accumulation by up to 81% in maize kernels under field conditions. The implementation of this macroporous starch polymer-based formulation, in combination with the biological control agent *A. flavus* AFCHG2, would not only reduce aflatoxin contamination in maize kernels but also minimise the use of food-grade seeds and grains for industrial purposes, thereby preserving their availability for human and animal nutrition. Consequently, this development could enhance the availability of these substrates for food and feed use, thereby contributing to improved safety and food security.

## 1. Introduction

Food safety is one of the most urgent global challenges in current times [1]. Since the world population will multiply in the next decades, the demand for resources will increase significantly, and we will need more quantity and higher quality of feed and food [2]. Additionally, the world will have to face the consequences of climate change that will affect the functioning of ecosystems, which directly and indirectly involve human and animal health as well as the yield of crops and food security [3].

Aflatoxins (AFs) are secondary metabolites produced by toxigenic strains of *Aspegillus flavus* and *A. parasiticus*, which can infect maize and other crops at different stages of the food chain and, under favourable conditions, synthesise these toxins. Among AFs, aflatoxin B_1_ (AFB_1_) is the most relevant due to its high prevalence and toxicity. In humans, AFB_1_ exposure may cause non-specific symptoms or progress to hepatocellular carcinoma [4]. In animals, AFB_1_ alters intestinal microbiota, induces immune and oxidative stress responses, damages intestinal integrity, and ultimately compromises nutrient absorption and growth performance [5]. In response to these risks, many countries have established and implemented regulatory limits for AFs in food, feed, and raw materials to safeguard consumer health [6,7,8,9]. Consequently, the agricultural sector experiences substantial economic losses due to the rejection of products that do not comply with these standards [10].

Maize (*Zea mays*. L) is one of the most important cereals at a global level. It is widely cultivated and used in several industries, such as food and beverage, and animal feed [11]. However, according to the latest DSM-Firmenich report [12], approximately 30% of maize kernels analysed worldwide in 2024 were contaminated with AFs, with average concentrations ranging from 4 to 62 μg/kg and a maximum detected level of 752 μg/kg. These values are of concern, particularly in light of the strict regulations established by the European Commission, which set maximum limits of 5 μg/kg for AFB_1_ and 10 μg/kg for total aflatoxins in maize and maize-derived products [9]. Given this concerning scenario, there is a pressing need to develop effective strategies to prevent and mitigate AF accumulation in this crop. For decades, several approaches have been performed in the frame of integrated pest management. To date, biological control, particularly involving non-toxigenic strains of *A. flavus*, is the most promising method to prevent AF contamination in cereal crops [13]. Indeed, various studies have demonstrated the successful application of formulations based on different non-toxigenic strains across multiple crops, including maize [14,15,16,17,18,19,20,21,22,23,24,25]. Even more, some of these formulations are currently commercialised [26]. In most of those formulations, the biocontrol agents have been grown on seeds and grains of different cereals such as rice, barley, and wheat. Nevertheless, three main factors have driven the replacement and optimisation of the matrix used to cultivate and deliver the biocontrol agent to the field: the physiological state of the agent, the strain’s ability to establish in the ecosystem, and the fact that seeds and grains are primarily intended for human and animal consumption. Thus, their use in bioformulate production would reduce their availability as food and feed. In this context, several experiments have been conducted over the years, taking into account the nutritional stability of the biocontrol agents and the low cost of production, storage, transportation, and application. Some examples are preparations based on alginate formulations [27], bioplastics [28,29], water-dispersible granules with tapioca starch [30], and reused plastic wastes [31,32,33]. Additionally, newer biocontrol strategies using dry spores of non-toxigenic *A. flavus* strains have been developed [34].

The research group of the Instituto de Investigación en Micología y Micotoxicología (IMICO, CONICET-UNRC) from Argentina has been working on biocontrol of AFs in peanut and maize crops since 2001, when the non-toxigenic *A. flavus* AFCHG2 strain was first isolated [35]. This strain was characterised morphologically and molecularly in addition to being evaluated as a potential biocontrol agent under in situ assays, field trials, and storage conditions as single and mixed inocula in combination with other potential biocontrol agents, demonstrating a reduction in AF contamination of up to 85–86% in comparison with non-inoculated controls [20,21,36,37]. Along with these experiments, formulations based on this non-toxigenic *A. flavus* strain were prepared using long-grain rice.

In line with the 17 Sustainable Development Goals of the United Nations [38], which include eradicating hunger and achieving food security, this study aimed to contribute to more sustainable food production systems. Specifically, the objective was to replace the seeds and grains used in current formulations by developing and evaluating, at the field stage, a macroporous starch polymer as a substrate and carrier of a biological control agent for AFs to be applied in maize fields.

## 2. Results and Discussion

### 2.1. Bioformulate Production Based on Macroporous Starch Polymer

Natural, economical, and starch-rich substrates were evaluated as alternatives to long-grain rice, which had been used as the substrate and carrier of the biocontrol agent in the previous formulation. Gelation represented a critical initial step in polymer formation, as it promotes starch matrix swelling and molecular reorganisation, enabling the establishment of a continuous polymeric network. This structural transition governs key physicochemical properties of the matrix, including homogeneity, stability, and processability. Gelation was induced by heating on a hot plate, and the temperature required was dependent on the starch source but independent of starch concentration (5, 10, or 15%), indicating that the thermal behaviour of the system is primarily dictated by the starch source. Proper control of this step was therefore essential to ensure a reproducible matrix formation and to support subsequent biotechnological processes, such as inoculation and conditioning, ultimately influencing the performance of the final bioformulated product (Table 1).

The gels were frozen, and the formation of ice crystals generated pores in the starch-based matrix. Afterwards, they were thawed, allowing water to drain from the pores. Thawing of the gels, whether performed at room temperature (approximately 20 °C) or accelerated in a forced-air oven, did not affect pore size. This indicates that the thawing rate had little influence on the structural characteristics of the starch-based matrix. Pore diameters varied within the same gel and among gels prepared with different starch sources and concentrations, as detailed in Table 1.

Although the pore size obtained with the tapioca- and rice starch-based polymers did not meet the desired pore diameter, the preparation of the bioformulates was continued to evaluate fungal development. Once the *A. flavus* AFCHG2 strain was inoculated, and after the incubation period, the tapioca- and rice starch-based polymers showed a viscous mass, lacking the consistency required to serve as an effective carrier for the biocontrol agent. A similar effect was observed in all formulations prepared with tapioca, rice, or maize starch when citric acid was omitted, as well as in those prepared with citric acid but without the curing step. Consequently, and considering that the largest pores were observed in the polymer prepared with 10% maize starch, this treatment was selected for the bioformulate preparation.

For the drying stage, it was necessary to obtain small pieces of the polymer by cutting it with a scalpel. After that, several oven sterilisation and curing procedures were assessed. Treatments below 100 °C for 10–15 min proved ineffective, as microbial contamination persisted in the polymer, indicating inadequate sterilisation. In contrast, temperatures above 100 °C led to caramelisation in all glucose-containing formulations, diminishing the availability of the carbon source. Optimal results were obtained with the polymer composed of 10% maize starch and 3% sucrose (instead of glucose), when treated at 110 °C for 15 min. Under these conditions, complete sterilisation was achieved, and citric acid (0.4–0.5%) induced starch cross-linking, imparting greater rigidity to the matrix, a property that remained in the final product.

Additional volumes of PBS and sterile distilled water, in the same proportion, were added to hydrate the polymer. This hydration corresponded to an a_w_ value of approximately 0.979, which was necessary to support adequate development of the biocontrol agent. Simultaneously with this hydration, an appropriate volume of NaOH (1M) solution was added. To optimise substrate utilisation and promote faster synthesis and release of amylase enzymes by the fungal strain, part of the sterile distilled water was replaced by a soluble starch solution (0.1, 0.25, and 0.5%). However, no visible differences in fungal growth were observed during the incubation period among the different starch concentrations or compared with the control without soluble starch. Consequently, the addition of soluble starch was excluded from the process in order to reduce manufacturing costs.

**Figure 1:**
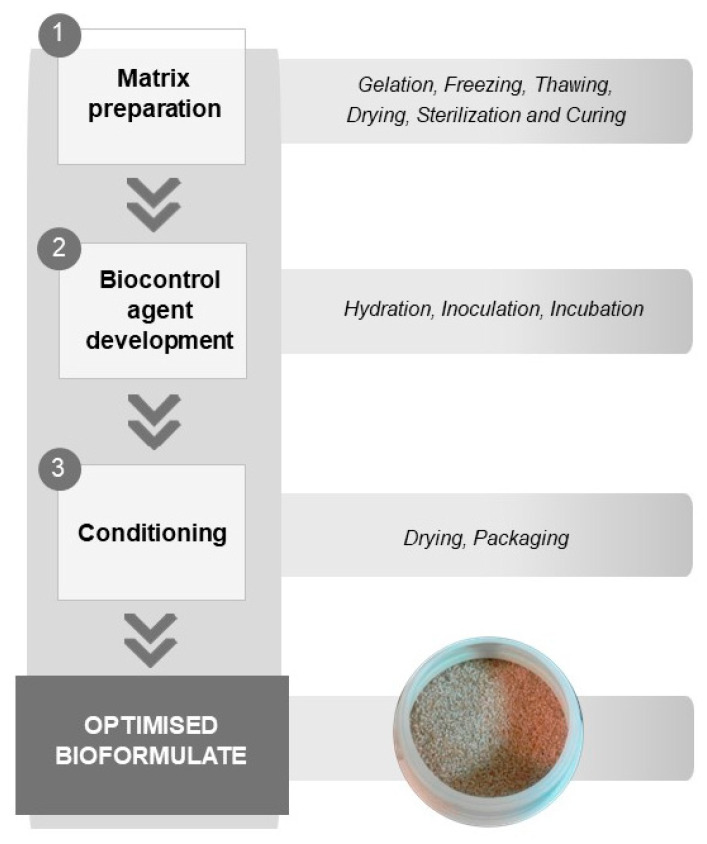
Conceptual workflow of the optimised bioformulation process, highlighting the main stages involved in matrix preparation, biocontrol agent development, and final conditioning steps.

Summarising the previous results, the optimal procedure included the steps described in Figure 1. The optimised synthesis process involved heating the mixture of all components and distilled water to 93–97 °C under constant stirring, followed by cooling, freezing, thawing, and drying. Subsequent sterilisation and curing at 110 °C for 15 min produced a stable polymer, which was then hydrated and adjusted to pH 6–7. After inoculation, the biocontrol agent colonised the macroporous matrix within 3–7 days, yielding a dry, storable product suitable for field application.

The procedure and product of this development led to a patent application submitted to the Instituto Nacional de la Propiedad Industrial, Argentina, with the web reference number 1682840.

**Figure 2:**
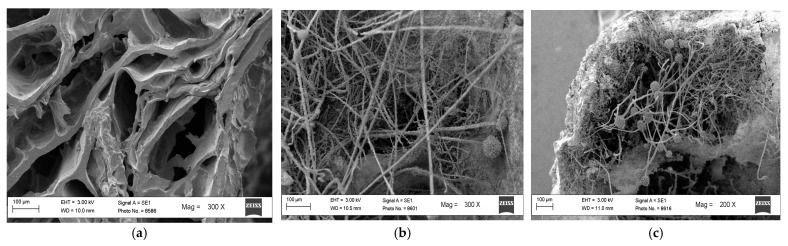
SEM micrographs of the developed starch-based biopolymer. (**a**) Polymer matrix in the absence of the biocontrol agent, showing a uniform macroporous structure generated by the freezing–thawing process; (**b**,**c**) polymer inoculated with *A. flavus* AFCHG2 after four days of incubation, displaying extensive hyphal growth and sporulation within the pores. Fungal mycelia and aspergillar heads are visible at 200× and 300× magnification, confirming active colonisation of the matrix.

To characterise the macroporous architecture of the synthesised polymer and to evaluate its suitability as a support for fungal growth, SEM imaging was performed under two conditions: in the absence and in the presence of the biocontrol agent. In the uninoculated polymer, a well-defined porous network was observed, confirming the effectiveness of the freezing–thawing process in generating macropores. When inoculated with *A. flavus* AFCHG2 and incubated for 4–5 days, the fungal hyphae were found to colonise the inner surfaces of the pores, indicating that the polymer structure provides a favourable microenvironment for the growth and development of the biocontrol strain (Figure 2).

Previous studies have developed carrier-based formulations for biocontrol, including commercial products such as Afla-Guard and Aflasafe in the United States and Africa. These typically rely on cereal grains rather than biopolymers as carriers [39,40,41]. More recent strategies employ bioplastics [42,43] or inert materials such as clays, vermiculite, or starch-based matrices [44,45,46]. Nonetheless, their production processes and physicochemical properties differ substantially from the approach proposed in the present study.

The US6306386 patent also employs non-toxigenic *A. flavus* strains for competitive exclusion, but its formulation relies on vegetable oil and silicate materials, which do not act as nutrient sources [39]. Similarly, a water-soluble granule formulation has been reported, though it was designed for spray applications [47]. In contrast, the present work proposes a solid product compatible with conventional agricultural machinery widely used in Argentina.

Other inventions have focused on carriers that transport the biocontrol agent without serving as substrates for fungal development, including joint efforts by Italian and American research groups. However, these approaches rely on the reuse of plastic residues rather than the fabrication of a biopolymer [31,32,33].

The most comparable precedents to the present work are formulations in which a bioplastic functions both as a carrier and as a nutrient source for biocontrol spores. One such material, Mater-Bi (Novamont), consists of starch-based bioplastics combined with other polymers, including starch, polycaprolactone, and small amounts of natural plasticizers. Related patents [48,49] describe starch-synthetic polymer composites applied in fields other than agriculture. Despite these similarities, the biopolymer developed in the present study is chemically and structurally distinct. Additional inventions have reported starch-based biopolymers for unrelated applications, such as drug delivery systems, including ES2824832T3, which describes an aqueous dispersion of destructured starch complexed with various polymers [50].

### 2.2. Field Trials

#### 2.2.1. Soil Analysis

In the present study, the efficacy of two formulations consisting of the previously tested long-grain rice formulation (RBF) and a novel macroporous starch-based biopolymer (biopolymer-based formulation, BBF) was evaluated for the control of native toxigenic *A. flavus* and the reduction in AFB_1_ accumulation in maize over three growing seasons (2019/2020, 2021/2022, and 2022/2023). A summary of the results are shown in Table 2.

In soil samples collected before the application of the formulations and at harvest, total filamentous fungal counts ranged from 10^4^ to 10^5^ CFU/g across all growing seasons, although significant differences were observed among plots at each sampling time, as well as between the two sampling times for each plot individually. A previous study conducted in the same geographical region reported a maximum value of 7.1 × 10^5^ CFU/g of soil [51], and Benito et al. [52] found a mean of 3.4 × 10^4^ CFU/g in soil collected from the southern region of Córdoba province. This is consistent with the results obtained in the present work. Fluctuations among plots detected at the first sampling time in each growing season were likely related to intrinsic agroecosystem factors, such as nutrient composition and availability (C and P), which influence the structure of microbial communities [53,54]. When total fungal counts were compared between pre-application and harvest times, increases of 17–54% were observed in the 2019/2020 and 2021/2022 seasons, with the exception of the RBF plot in 2021/2022. However, these increases are unlikely to be related to the bioformulates, as substantial reductions in total fungal counts were recorded in 2022/2023 and in the RBF plot of the 2021/2022 season. Alternatively, the presence of inhibitory compounds affecting certain microbial groups associated with the maize phenological stage could explain the variations observed in soil samples collected at harvest [55]. Moreover, seasonal and climatic variations may also play a key role in shaping soil microbial dynamics [56].

Regarding the relative density of *A. flavus* in soil samples, significant increases were observed in the treated plots during the 2019/2020 and 2021/2022 seasons, whereas no significant differences were detected between sampling times in the control plots of these field trials. This increase in *A. flavus* density is consistent with successful establishment and local multiplication of the applied non-toxigenic *A. flavus* AFCHG2 strain, a pattern reported in multiple field studies where carrier-based non-toxigenic formulations increased non-toxigenic frequencies and reduced AFB_1_ contamination [57,58]. In contrast, during the 2022/2023 season, no significant differences were observed within plots when comparing both sampling times. This result could be explained by climatic conditions that limited the establishment or growth of the applied strain. For instance, lower temperatures or below-average rainfall during specific periods of the growing season may have restricted its development. Reduced persistence or insufficient release of propagules from the bioformulates could also have played a role. In addition, antagonism or competition from the resident soil microbiota may have further constrained the strain’s effectiveness.

The isolation frequency of toxigenic *A. flavus* varied among plots within the same field trial and soil sampling time, as well as among plots receiving the same treatment across different growing seasons. During the 2019/2020 season, both treated plots exhibited a decrease (20–58%) in the proportion of toxigenic isolates at harvest time, which is consistent with the application of the bioformulates. Nevertheless, in soil samples from treated plots in subsequent seasons, the percentage of toxigenic isolates either increased or showed no significant differences compared with the initial sampling (RBF, 2021/2022 season). As previously discussed for total fungal communities, similar factors also shape *A. flavus* colonisation and dynamics. Climatic conditions and soil properties further modulate its relative abundance and toxigenic potential [59]. In the present study, agricultural practices were the same across the three growing seasons. Thus, the observed increases or lack of reduction in toxigenic isolates in some treated plots across seasons could reflect a combination of crop phenology and local edaphoclimatic conditions rather than the direct effect of the bioformulates. Despite these considerations, the frequency of toxigenic isolates was significantly lower in the treated plots (17–35%) than in their corresponding controls (50–60%). This could be indicative of the different AFB_1_ levels expected in maize kernels.

In summary, the observed patterns in soil samples indicate that *A. flavus* AFCHG2 can establish within the soil microbiota. A study conducted in the United States, in which three non-toxigenic *A. flavus* strains were applied to commercial maize fields, showed that two months after application, the number of toxigenic strains in soil decreased from 48% to 9% [60]. In brief, the non-toxigenic biocontrol strains persisted in the ecosystem, similar to the strain used in the present study and grown on the two different formulations. Although there were some significant differences between RBF and BBF, the behaviour of the biocontrol agent was independent of the substrate in which the *A. flavus* strain was developed. However, the competitive performance of *A. flavus* AFCHG2 in the present study appears to depend on other conditions. Therefore, kernel analyses are essential to further assess the efficacy of this biocontrol strategy.

#### 2.2.2. Maize Kernel Analysis

The results of *A. flavus* prevalence, aflatoxigenic isolate percentages, and AFB_1_ contamination levels in maize kernels from all the field trials are shown in Table 2.

In samples from the 2019/2020 and 2021/2022 growing seasons, the treated plots showed a significantly higher prevalence of *A. flavus* compared with the respective controls. This could be attributable to the effect of the biocontrol agent applied through the bioformulates. Even more, this greater incidence was associated with a lower frequency of toxigenic isolates in the treated plots, in comparison with each control. Similarly, in maize kernels collected from the field trial conducted during the 2022/2023 season, the percentage of aflatoxigenic isolates was significantly higher in the control plot than in the treatments with any of the bioformulates under study. Unlike the 2019/2020 and 2021/2022 growing seasons, the last field trial showed a higher *A. flavus* prevalence in the control plots than in the treated ones, with most isolates being aflatoxigenic (67%). Such variability in the infective performance of the biocontrol agent likely reflects season-to-season differences in environmental and crop-related conditions [61,62,63].

Regarding AFB_1_ incidence, the differences observed in kernel population structure between treated plots and their respective controls in the 2019/2020 and 2022/2023 seasons were associated with reduced AFB_1_ levels in kernels from the treated plots. In those seasons, AFB_1_ concentrations ranged from 4.0 to 10.9 μg/kg in treated plots, whereas significantly higher levels (27.5 and 32.3 μg/kg) were detected in the untreated plots. In these two growing seasons, the reduction in AFB_1_ contamination ranged from 66 to 85%, and the maximum effectiveness of the BBF was 81%. These results suggest that the applied biocontrol agent remained viable until harvest in both bioformulates, successfully competed with native toxigenic strains, and colonised the kernels, thereby preventing AFB_1_ production.

During the 2021/2022 growing season, no AFB_1_ was detected in any of the plots, which may be attributed to the absence of meteorological conditions conducive to AF synthesis. According to the Servicio Meteorológico Nacional Argentino (SMN, [64]), rainfall in this agricultural region was below the historical average during that season; however, maximum temperatures were around 26 °C. The temperature accepted as the optimum for AF synthesis is 28 °C [65,66], and the AF gen cluster expression may be influenced by other parameters such as substrate, CO_2_ concentrations, and a_w_ [67]. In addition to temperature, such conditions may have been unlikely to have favoured AFB_1_ production by native toxigenic *A. flavus* strains. In contrast, during the 2019/2020 and 2022/2023 growing seasons, maximum recorded temperatures averaged around 29 °C, with peaks reaching up to 36 °C in December 2022, creating conditions conducive to AFB_1_ production.

The results obtained across the three growing seasons show that the tested bioformulates modified total fungal abundance in soil, effectively displaced aflatoxigenic *A. flavus* strains in both soil and kernels, and significantly reduced AFB_1_ levels in harvested maize kernels. Both formulations (the long-grain rice carrier and the macroporous starch-based biopolymer) supported the survival and functionality of the biocontrol agent, exhibiting comparable performance. Although rice is a well-established carrier for non-toxigenic *A. flavus* strains [21,36], its use raises concerns related to food safety and increasing global demand. In this context, the starch-based biopolymer emerges as a sustainable and scalable alternative, demonstrating efficacy in preventing AFB_1_ accumulation in maize kernels and positioning itself as a promising tool for large-scale AF biocontrol. Notably, its formulation relies on non-nutritional-grade starch, ensuring that it does not compete with starch supplies intended for human or animal consumption.

## 3. Conclusions

A novel macroporous starch-based bioformulate carrying a non-toxigenic *Aspergillus flavus* strain was developed as a low-cost and sustainable carrier material for the growth and delivery of a biological control agent. This represents the first bioformulation produced using a non-toxigenic *A. flavus* strain supported on a starch polymer matrix. A product and process patent application related to this technological development has been submitted to the Instituto Nacional de la Propiedad Industrial (Argentina).

Under field conditions, the starch-based bioformulate effectively reduced aflatoxin B_1_ (AFB_1_) contamination in maize kernels by up to 81%, showing performance comparable to that of the same biocontrol strain formulated on traditional long-grain rice. These results demonstrate that the novel formulation matches the efficacy of conventional carriers while offering significant economic and environmental advantages.

Overall, this innovation provides an environmentally friendly and economically viable alternative for aflatoxin management, contributing to the production of safe food and feed and strengthening the agro-productive sector from both economic and sanitary perspectives. Furthermore, it aligns with the United Nations Sustainable Development Goal of achieving “Zero Hunger” by promoting safer, more sustainable, and accessible agricultural systems.

## 4. Materials and Methods

### 4.1. Fungal Strain

The non-toxigenic strain *A. flavus* AFCHG2 was used in the present studies as the biocontrol agent. It was collected from Córdoba province, Argentina, during 2001 [35] and described as a non-producer of neither AFs nor cyclopiazonic acid. This strain produces sclerotia > 400 μm (morphotype L) and belongs to a vegetative compatibility group (VCG) that only includes non-toxigenic strains [68,69,70]. Additionally, it has shown significant reductions in AF accumulation under field and storage conditions when formulated on long-grain rice, making it a good candidate as a biocontrol agent [21]. *Aspegillus flavus* AFCHG2 belongs to the culture collection of the Instituto de Investigación en Micología y Micotoxicología (IMICO, CONICET-UNRC), Argentina.

### 4.2. Preparation of the Novel Bioformulate

#### 4.2.1. Composition of the Bioformulate

Two different formulations were prepared for field assays. One of them was based on solid fermentation of long-grain rice, as it was described by Alaniz Zanon et al. [36]. The novel formulation was developed with the aim of obtaining a mesostructured material composed of solid particles with significant macroporosity that supports fungal growth within the solid matrix. Part of these assays were conducted in the Instituto de Investigaciones en Tecnologías Energéticas y Materiales Avanzados (IITEMA, CONICET-UNRC). Starch, a biodegradable and low-cost polymer, was employed as the structural component [71]. Therefore, macroporous starch-based polymers were prepared using various industrial-grade starches (tapioca, rice, or maize), selecting only one source per formulation. These starches were not suitable for food or feed manufacturing but were intended for industrial applications such as adhesives or paint thickeners. The starch concentration in the formulations was 5, 10, and 15% for maize starch, and 10 and 15% for tapioca and rice starch. Additional components included urea (0.2–0.5%) as a nitrogen source, glucose and sucrose (2–3%) as carbon sources, and citric acid (0.3–0.6%), a biocompatible compound that produces starch chain covalent cross-linking and enhances the gel’s mechanical properties [72]. All these compounds were added to distilled water.

Considering that the *A. flavus* strain may require an available soluble starch fraction to induce amylase synthesis and support growth [73], soluble starch solutions were prepared in distilled water at concentrations of 0.1, 0.25, and 0.5% to replace the distilled water used during inoculation. To prepare these solutions, the appropriate amount of soluble starch was weighed, mixed with distilled water at room temperature to form a paste, and then gradually added to the remaining volume of boiling water. Once homogeneity was achieved, the solution was sterilised by autoclaving (121 °C, 20 min) and it was stored at 4 °C.

#### 4.2.2. Synthesis and Characterisation of the Macroporous Polymer

The procedures used for the production of the macroporous starch polymer were adapted from Zhang et al. [74], Liu et al. [75], and Zhou et al. [76,77]. Polymer preparation included steps such as gelation, freezing, thawing, drying, sterilisation, curing, hydration, and pH adjustment.

Among the procedures tested, starch gelation at moderate temperatures (>50 °C) was employed to induce gel formation, with the exact temperature depending on the starch type, particularly its amylose content [78]. Several time and temperature combinations were tested to optimise the synthesis process. Porosity was assessed by staining the gels with iodine vapours. Briefly, iodine granules were placed in a Petri dish together with small pieces of each gel. Once the gel fragments turned purple, they were removed, sectioned, and mounted on a glass slide with a drop of water. The samples were covered with a coverslip and examined under a light microscope at 400× magnification (Carl Zeiss, SMD F6D, Germany). Additional parameters, such as pH, were also determined.

#### 4.2.3. Inoculation and Growth of the Biocontrol Agent

Once the polymer was synthesised and dried, it was properly rehydrated. To enable fungal growth, the pH was adjusted by adding a NaOH solution (1M) and a potassium phosphate buffer (PBS) 1X, since citric acid lowers the solution pH. Water activity (a_w_) was determined with an AquaLab Series 3 (Decagon Devices Inc., Pullman, WA, USA) at 20 °C (room temperature). The gel was inoculated following the same procedure used for the long-grain rice formulation. Briefly, a conidial suspension of the *A. flavus* AFCHG2 (10^6^–10^8^ conidia/mL) was used for inoculation. The incubation was performed at 28–30 °C for 3–7 days. The substrate was dried in a forced-air oven, and the viable count (CFU/g) of *A. flavus* was confirmed. Both bioformulates were stored at room temperature until field application in maize crops.

#### 4.2.4. Scanning Electron Microscopy (SEM) Analysis

The samples were examined under a scanning electron microscope (Carl Zeiss EVO MA10, Carl Zeiss Microscopy GmbH, Oberkochen, Germany). Prior to imaging, samples were dried on a stove for 24 h and then dehydrated for 96 h in a silica-gel desiccator and subsequently sputter-coated with a thin layer of gold to prevent charging artefacts.

### 4.3. Field Assay Design

Field assays were conducted during 3 maize growing seasons: 2019/2020, 2021/2022, and 2022/2023, in commercial fields from Córdoba province, Argentina. The trial consisted of a randomised block design with three repetitions. Each treatment and control plot consisted of 30 m × 10 m, divided into three 10 m × 10 m subplots, with buffer areas among plots of 5 m. The maize kernels (Nidera Ax 887 HCL-MG for season 2019/2020, and Nidera 7921 CL Viptera, 3 types, for seasons 2021/2022 and 2022/2023) were planted in rows at 50–52 cm distance each. Subplots included controls and treatments as follows: non-inoculated control; inoculation with bioformulate based on *A. flavus* AFCHG2 strain growth on long-grain rice (rice-based formulate, RBF); and inoculation with bioformulate based on *A. flavus* AFCHG2 strain growth on the macroporous starch polymer (biopolymer-based formulate, BBF). The bioformulates were manually applied at a rate of 30 kg inoculum/ha within the V4–V6 maize phenological stage.

### 4.4. Total Fungal and Aspergillus Section Flavi Count in Soil and Maize Kernel Samples

Soil samples were collected from each subplot to determine total fungal and *Aspergillus* section *Flavi* counts at 2 stages: Previous to the bioformulates application and at harvest time. For this, 5 soil samples were taken in two diagonal transects extending from opposing corners in each subplot [20]. Each soil sample was a pool from 5 sub-samples taken from the top 5 cm of soil, which were combined in a paper bag and air-dried for 1–2 days at 25–30 °C. From each soil sample, 10 g were used to prepare decimal dilutions that were spread on Dichloran Rose Bengal Chloramphenicol (DRBC) agar. The plates were incubated in the dark for 7 days at 28 ± 1 °C. Data were expressed as colony-forming units per gram of soil (CFU/g). Fungal colonies coincident with typical characteristics of *Aspergillus* section *Flavi* were sub-cultivated in malt extract agar (MEA) for later identification [79]. Relative densities of *A. flavus* were calculated using the following equation [80]:

(1) $$\mathrm{Relative}\;\mathrm{Density}\;(\%)\;=\;\frac{(\mathrm{Number}\;\mathrm{of}\;A.\;\mathrm{flavus}\;\mathrm{isolates}\;\times \;100)}{\mathrm{Total}\;\mathrm{number}\;\mathrm{of}\;\mathrm{isolates}\;\mathrm{from}\;\mathrm{all}\;\mathrm{genera}\;\mathrm{or}\;\mathrm{species}}$$

Additionally, the toxigenic ability of a representative number of isolates from soil samples (25–30% of the isolated *A. flavus* from each plot) was evaluated according to Alaniz Zanon et al. [36].

Regarding kernel analysis, during harvest seasons, around 30 ears from each subplot were manually collected following a “W” design. Samples were manually husked and mechanically shelled, and kernels were superficially disinfected according to Pitt and Hocking [81]. Briefly, 100 kernels of each subplot were immersed in 70% ethanol for 2 min, followed by 0.4% chlorine for 2 min, and rinsed with sterile distilled water. Kernels were directly plated on dichloran glycerol 18% agar (DG18) and incubated at 28 ± 1 °C for 7 days [37]. Morphological identification was conducted according to Klich [79], and relative densities of *A. flavus* were estimated as previously described [80]. Toxigenic potential of isolates from kernel samples was assessed [36].

### 4.5. Aflatoxin Determination in Maize Kernels

Aflatoxin B1 was detected and quantified in maize kernels from all treatments and control plots. The AFB1 extraction was performed in triplicate following a Quick, Easy, Cheap, Effective, Rugged, and Safe extraction (QuEChERS) procedure [82,83]. Samples were finely ground, and 2.5 g were mixed with 7.5 mL of a solvent mixture and shaken. After that, 0.45 g of anhydrous sodium acetate and 1.15 g of anhydrous magnesium sulphate were added and stirred at each step. The mixture was centrifuged and maintained overnight at −20 °C. Four millilitres of the supernatant were recovered and evaporated. Aflatoxins were detected and quantified using HPLC (Waters Alliance e2695 HPLC system, Waters Corporation, Milford, MA, USA), according to Alaniz Zanon et al. [21]. Reference AFB1 standard was used (Sigma-Aldrich, St. Louis, MO, USA), and the limit of detection was 1 μg/kg.

### 4.6. Statistical Analysis

Data on fungal populations were log-transformed prior to analysis of variance (ANOVA). Mean separation and comparisons were made by Fisher’s least significant difference (LSD) test at a probability level of *p* < 0.05. To compare the different treatments within a field trial, AFB_1_ concentrations were subjected to a nonparametric Kruskal–Wallis test followed by Dunn’s nonparametric multiple comparison test. The statistical analyses were performed using InfoStat, 2010 [84].

## 5. Patents

Patent application with the web reference number INPI (Instituto Nacional de la Propiedad Industrial, Argentina) 1682840 entitled “Bioinsumo basado en un biopolímero macroporoso de almidón y su procedimiento de fabricación”, filed by Sofía Noemí Chulze, César Alfredo Barbero and María Silvina Alaniz Zanon (filed 2 March 2022; pending). The inventors overlap with some of the authors of this manuscript, and the application is related to the experimental procedures described in the present manuscript.

## Figures and Tables

**Table 1:** Characteristics of the different macroporous polymers according to the starch source.

Starch Source	Starch Proportion (%)	Media Gelation Temperature (°C)	Media Porous Diameter (µm)
Tapioca	10	56 ± 2	42.2 ± 13.2
	15	57.5 ± 2	68.8 ± 23.3
Rice	10	88.5 ± 1	67.2 ± 15.7
	15	90 ± 2	72.5 ± 34
Maize	5	94 ± 1	53.7 ± 25.2
	10	94 ± 0.5	96.15 ± 25.9
	15	95 ± 1.5	64.9 ± 13.9

Results shown in this Table consider gel composition of each of the mentioned starch sources (tapioca, rice, and maize) in the respective proportions (5, 10, or 15%), with the addition of 3% sucrose, 0.3% urea, and 0.5% citric acid.

**Table 2:** Summary of fungal incidence, *Aspegillus flavus* population, aflatoxigenic ability, and aflatoxin B_1_ levels in the three field trials.

			Growing Season and Treatments								
			2019/2020			2021/2022			2022/2023		
			RBF ^a^	BBF ^b^	Control	RBF	BBF	Control	RBF	BBF	Control
Soil	Pre- treatment stage	Total fungal count (CFU/g)	4.1 × 10^5^ a	7.4 × 10^5^ c	3.4 × 10^5^ a	1.4 × 10^5^ c	8.8 × 10^4^ b	9.7 × 10^4^ b	1.1 × 10^5^ b	3.1 × 10^5^ d	1.8 × 10^5^ c
		Relative density of *A. flavus* (%)	2.4 b	1.3 a	4.6 d	9.9 c	ND ^c^ a	60.8 e	7.1 b	5.4 a	5.3 a
		Aflatoxigenic isolates (%)	50 d	25 c	17 a	19 b	ND a	23 c	ND a	ND a	34 c
	Harvest stage	Total fungal count (CFU/g)	8.4 × 10^5^ d	8.9 × 10^5^ d	5.6 × 10^5^ b	4.1 × 10^4^ a	1.9 × 10^5^ d	1.4 × 10^5^ c	2.4 × 10^4^ a	2.6 × 10^4^ a	3 × 10^4^ a
		Relative density of *A. flavus* (%)	5.5 e	3.7 c	2.2 b	10.2 d	4.2 b	3.6 b	7.5 b	6.0 a	4.8 a
		Aflatoxigenic isolates (%)	21 b	20 b	50 d	17 b	25 c	56 d	35 c	7 b	60 d
Kernels	*A. flavus* prevalence (%)		24 c	12 b	9 a	8 b	11.5 c	4.5 a	0.5 a	1.5 b	4.5 c
	Aflatoxigenic isolates (%)		21 a	25 a	78 c	33 a	32 a	43 b	ND a	ND a	67 b
	Aflatoxin B_1_ (μg/kg) ^d^		4.0 ± 2.3 a	5.2 ± 2.4 a	27.5 ± 9.2 b	ND	ND	ND	5.4 ± 1.6 a	10.9 ± 1.2 ab	32.3 ± 5.5 b

Statistical analyses were performed by grouping all determinations of the same type within each field trial. Different letters show significant differences between treatments (*p* < 0.05). ^a^ RBF: Rice-based formulation; ^b^ BBF: Biopolymer-based formulation; ^c^ ND: Not detected; ^d^ LOD (AFB_1_): 1 μg/kg.

## Data Availability

The original contributions presented in this study are included in the article. Further inquiries can be directed to the corresponding author(s).

## Abbreviations

The following abbreviations are used in this manuscript:

AFs	Aflatoxins
AFB_1_	Aflatoxin B_1_
RBF	Rice-based formulate
BBF	Biopolymer-based formulate
ND	Not detected
LOD	Limit of detection
